# Seed Germination Ecology and Longevity of the Invasive Aquatic Plant *Sagittaria platyphylla*

**DOI:** 10.3390/plants14203138

**Published:** 2025-10-12

**Authors:** Nguyen Nguyen, Tobias Bickel, Sundaravelpandian Kalaipandian, Steve Adkins

**Affiliations:** 1 School of Agriculture and Food Sustainability, The University of Queensland, St Lucia, QLD 4072, Australia; s.kalaipandian@uq.edu.au (S.K.); s.adkins@uq.edu.au (S.A.); 2 Biosecurity Queensland, Department of Primary Industries, Brisbane, QLD 4102, Australia; tobias.bickel@dpi.qld.gov.au; 3 Queensland Alliance for Agriculture and Food Innovation, The University of Queensland, Indooroopilly, QLD 4068, Australia; 4 Department of Bioengineering, Saveetha Institute of Medical and Technical Sciences (SIMATS), Saveetha School of Engineering, Chennai 602105, Tamil Nadu, India

**Keywords:** seed longevity, delta arrowhead, water weed, seedling emergence, seedbank dynamics, sagittaria

## Abstract

*Sagittaria platyphylla* (Engelm.) J.G.Sm. is an invasive aquatic plant of concern in Australian freshwater systems. Understanding its seed germination ecology and seedbank longevity is critical for effective management. This study examined environmental influences on germination and longevity through three controlled experiments. Seeds germinated between 17 and 29 °C, with optimal germination (96 ± 2%) at 21 °C under a 12/12 h light/dark photoperiod. High germination (93–99%) also occurred under light in diurnal regimes of 15/5 °C, 25/15 °C, and 30/20 °C. In a burial experiment, seedlings emerged only from surface-sown seeds (76 ± 4%); no emergence occurred from buried seeds, though viability remained high, peaking at 98 ± 2% at 2.5 cm depth. A controlled aging test indicated a 50% viability loss (P_50_) in 36 days under warm, moist laboratory conditions. Based on established criteria, *S. platyphylla* produces short-lived seeds, which are likely to persist in the substrate seedbank for <1 to 3 years. The strong light dependence of germination suggests that sediment disturbance, which exposes buried seeds to light, could significantly enhance recruitment, highlighting the importance of minimizing disturbance for effective long-term management.

## 1. Introduction

Sagittaria or arrowhead [*Sagittaria platyphylla* (Engelm.) J.G.Sm.] is a highly invasive aquatic plant (IAAP) native to North America [1]. It has become naturalized and invasive in multiple regions globally, including in Australia, where it has been listed as a Weed of National Significance due to its severe ecological and economic impacts [2,3]. The species aggressively invades natural wetlands, irrigation channels, and drainage systems, forming dense monocultures that restrict water flow, degrade aquatic habitat quality, and outcompete native flora and fauna [4]. These dense stands pose a significant threat to biodiversity and waterway functionality; hence, *S. platyphylla* management is a priority in infested regions [5]. The success of *S. platyphylla* as an invader is largely attributed to its versatile reproductive strategies. It reproduces both asexually and sexually, allowing for rapid spread and population recovery after disturbance [1]. Asexual propagation occurs through fragmentation of shoots and the production of vegetative propagules such as rhizomes, tubers, and stolons [3]. In addition to asexual propagation, the species produces large numbers of viable achenes (hereafter referred to as seeds), with estimates of up to 20,000 seeds produced per plant during a single season [6,7]. Despite the emphasis often placed on vegetative reproduction in IAAPs, seed-based recruitment from the substrate seedbank (SSB) is also playing a major role in population persistence and re-establishment following control interventions [8,9].

Seed-based recruitment from the SSB can serve as a persistent source of reinfestation [10,11]. Germination is a key stage in the plant life cycle, directly influencing population establishment, spread, and persistence [12,13]. For invasive species like *S. platyphylla*, understanding the environmental triggers that promote or inhibit germination is crucial for developing targeted control strategies. For example, synchronized germination events may allow for the application of accurately timed application of control measures (e.g., herbicides) that target all seedlings simultaneously, potentially depleting the seedbank over time [14,15]. Such synchronous germination offers an opportunity for uniform and potentially permanent control of IAAPs [15]. Conversely, understanding dormancy mechanisms and inhibitory environmental conditions may help suppress seed recruitment and prevent spreading. However, germination of aquatic plants from the SSB is influenced by numerous environmental factors such as light intensity, water temperature, seed burial depth, and seed dormancy [16].

*Sagittaria platyphylla* seeds have been shown to germinate year-round under favorable conditions, with a preference for silty substrates over clay [1,17]. It is believed that *S. platyphylla* germination is accelerated under anaerobic conditions, a response that is common in other Alismataceae species [1]. Insights from related species support this view: for example, *S. latifolia* Willd. requires light for germination and performs best when seeds are located near the substrate surface [18]. Similarly, *S. trifolia* L. exhibits light and temperature-dependent germination, particularly under warm (30/20 °C) and hypoxic conditions, with delayed but increasing germination after extended periods on the substrate surface [19]. In addition, *S. lancifolia* L. has been observed to reach 100% germination under light at 25 °C [20]. High seedling emergence in *S. montevidensis* Cham. & Schltdl. has been reported from seeds buried at depths of 0.5 to 1.0 cm, indicating a tolerance for shallow burial depth [21]. These observations suggest that *S. platyphylla* germination may be affected by burial depth, light, and temperature, but these factors remain poorly studied.

In addition to seed germination ecology, seed longevity in the SSB is a fundamental aspect of population dynamics and management planning [22,23]. Seed longevity or lifespan refers to the duration of seeds remain viable [24]. It is an important aspect of IAAP management as eradication cannot be deemed successful if viable seeds remain in the SSB [25]. Seed longevity is a complex characteristic that varies widely across species and even among different seed lots within the same species [24]. Moisture and temperature are key environmental factors that determine seed survival or mortality, influencing the timescale over which lifespan is measured [26]. Data on seed longevity of aquatic plants are limited, but some studies on germination and seed persistence suggested that the seeds need a special mechanism to maintain their seed viability in the natural habitat through many seasons or for ex situ storage [27]. Some IAAP seeds can remain viable for many years if buried, such as *Pontederia crassipes* Mart., which has been shown to survive up to 28 years [28], and *Gymnocoronis spilanthoides* (D.Don) DC., which has a seed longevity up to 16 years [29]. In contrast, other aquatic plant species such as *Myriophyllum petraeum* Orchard, *Myriophyllum balladoniense* Orchard, *Glossostigma drummondii* Benth., and *Rithuria submersa* Hook.f. produce seeds that are all short-lived (1 to 3 years) in the seedbank [30].

Currently, no empirical data/studies exist on the longevity of *S. platyphylla* seeds in the SSB, representing a critical knowledge gap [1]. It is difficult to estimate how long sites remain at risk of reinfestation following treatment without the understanding of seed longevity. Traditional seed longevity studies rely on long-term field burial experiments [31], which are labor-intensive and time-consuming. A more rapid and widely adopted alternative is the controlled aging test (CAT) [25]. In this method, seeds are exposed to elevated temperature (45 °C ) and 60% relative humidity (RH) in a sealed chamber to accelerate the deterioration process [25]. At regular intervals, samples are removed and tested for germination to assess viability decline over time [25]. Although this method does not replicate natural conditions, it provides a practical and comparative approach for estimating relative longevity, particularly for invasive or problematic weed species where rapid data are needed [32]. Thus, this study aims to address critical gaps in our understanding of *S. platyphylla* seed ecology, focusing on factors that trigger germination and determine seed persistence in the SSB. This knowledge is essential for informing integrated control strategies that target both above-ground biomass and SSBs, thus reducing long-term reinvasion risks. Specifically, this study aims to (1) assess the influence of environmental variables (light, temperature, and burial depth) on germination, and (2) estimate seed longevity using a CAT.

## 2. Results

### 2.1. Seed Fill Analysis

**Figure 1:**
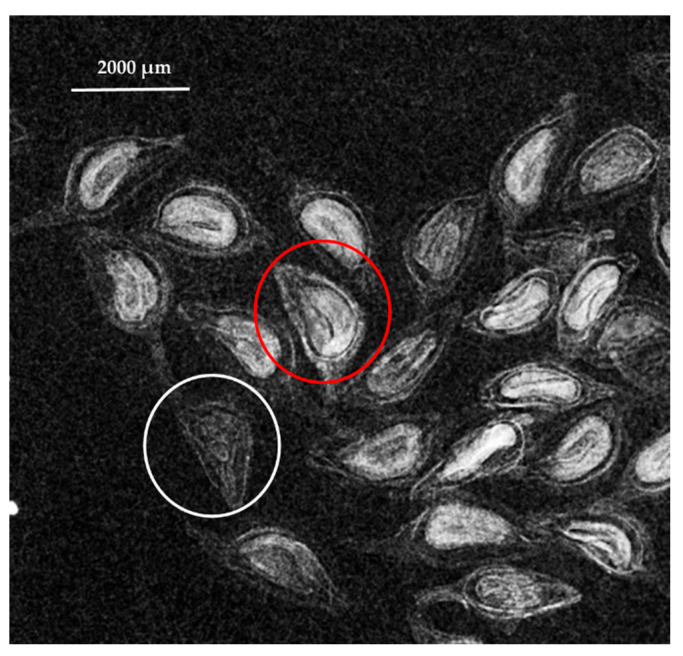
Seeds of *S. platyphylla* under X-ray examination (Faxitron MX-20), with the seed circled in red identified as filled and the seed circled in white considered to be unfilled.

The seed fill percentage was determined by analysis of the X-ray images, which revealed that the average seed fill percentage of the *S. platyphylla* seed lot was 95 ± 2%. Figure 1 presents a representative X-ray image from one replicate of the seed lot used to assess seed fill.

### 2.2. Experiment 1—Effects of Light and Temperature on Seed Germination

**Figure 2:**
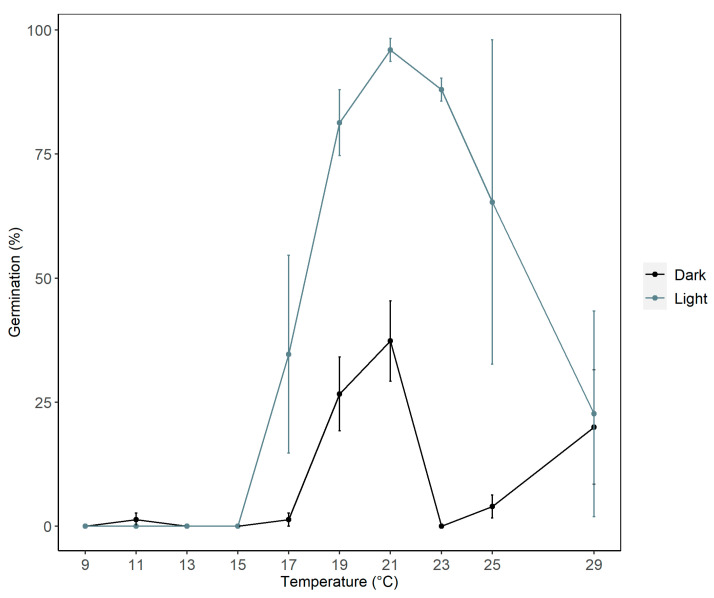
The 28-day germination percentage of *S. platyphylla* seeds under a range of constant incubation temperatures and using either a 12/12 h (light/dark) photoperiod or under constant darkness. The data are for the mean germination of three replicates of 25 seeds, and bars represent ± standard error.

A significant interaction was observed between incubation temperature and light conditions (Generalized Linear Model, GLM binomial, *p* < 0.0001, Figure 2). Germination predominantly occurred at warmer temperatures (17 to 29 °C) under both light and dark conditions, but with significantly higher germination rates under light (GLM binomial, *p* < 0.0001) (Figure 2). The average percentage germination achieved under both illumination conditions ranged from ca. 37 (dark) to 96% (light). Under light conditions, germination remained low at temperatures below 17 °C and then increased sharply, peaking at 21 °C with ca. 96 ± 2% (GLM binomial, *p* < 0.0001). Beyond 21 °C, germination declined. Under constant dark conditions, germination was low, below 17 °C, increased steadily between 17 °C and 21 °C (reaching 37 ± 8%), dropped to near zero at 23 °C, and then slightly increased again toward 29 °C (20 ± 11%). The results of a Type II analysis of deviance (ANOVA) are summarized in Table 1.

**Figure 3:**
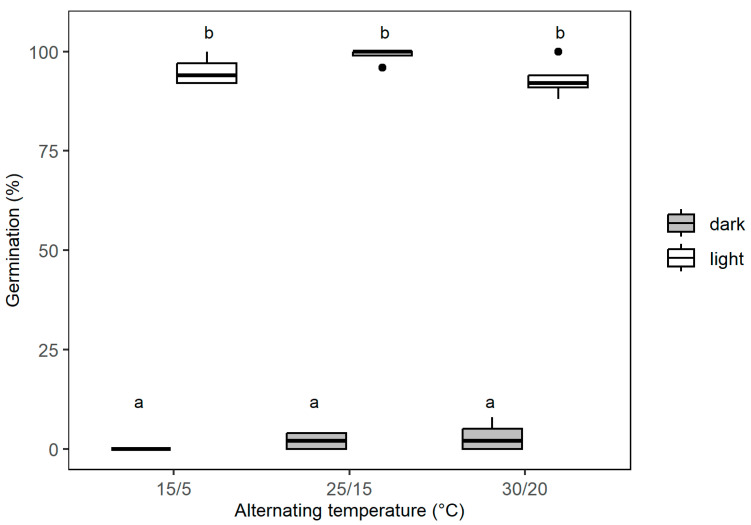
Effect of three different diurnal temperature regimes on germination of *S. platyphylla* seed under a 12/12 h (light/dark) photoperiod of white light and darkness after 28 days. The box plots span the 25th and 75th quartiles, the band in the box is the median, and the whiskers indicate the data range. Outliers are denoted as dots. Letters indicate the significant difference between the treatment means at *p* < 0.05 (TukeyHSD).

Under three contrasting alternating temperature regimes, the light/dark condition had a significant effect on seed germination (GLM binomial, *p* < 0.0001), while the effect was not significant for diurnally alternating temperature (GLM binomial, *p* = 0.14) and for the interaction between the two factors (GLM binomial, *p* = 0.06) (Figure 3). These results confirm that 24 h darkness significantly inhibits the germination of *S. platyphylla* seeds and does so across all three temperature regimes when compared to seeds exposed to light. Although temperature had no significant effect on germination, light had a substantial impact. Under the 12/12 h (light/dark) photoperiod, the mean germination rate across all three temperatures was ca. 96%, while in constant darkness, it dropped dramatically to just ca. 2% (Figure 3).

### 2.3. Experiment 2—Effects of Burial Depth on Seedling Emergence and Viability of Retrieved Seeds

**Figure 4:**
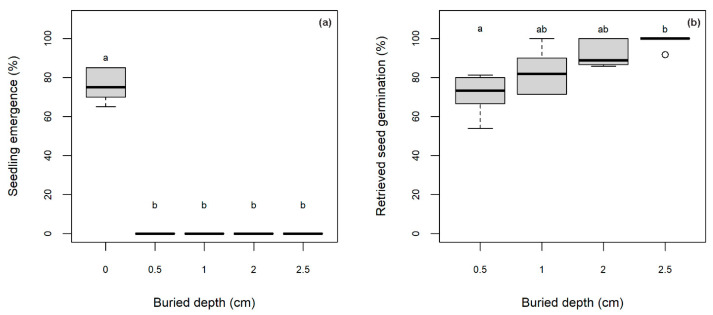
Effects of five different burial depths (0.0 to 2.5 cm) on *S. platyphylla* seedling emergence (**a**) and germination of retrieved seeds (**b**). In panel (**b**), all seeds from the substrate surface (non-buried treatment, 0 cm) had fully decomposed and could not be recovered. The box plots span the 25th and 75th quartiles, the band in the box is the median, and the whiskers indicate the data range. Outliers are denoted as dots. Letters indicate the significant difference between the treatment means at *p* < 0.05 (TukeyHSD).

Burial depth significantly inhibited seedling emergence as only non-buried seeds germinated (GLM binomial, *p* < 0.0001). Seedling emergence at the surface level reached 76 ± 4%, while there was no emergence recorded for the other four burial depths (Figure 4a). After 3 months, all remaining non-germinated seeds on the surface (non-buried) had decayed and could not be retrieved. Therefore, only the buried seeds were retrieved for viability testing. There was a significant difference in the germination of the retrieved seeds from the different burial depths (GLM binomial, *p* = 0.0007). The highest germination (98 ± 2%) was recorded for the seeds initially buried at 2.5 cm. However, this was not statistically different when compared to seeds buried at depths of 1.0 or 2.0 cm (83 ± 6% and 92 ± 3%, respectively; Figure 4b). At the 0.5 cm burial depth, the retrieved seeds showed the lowest germination percentage (71 ± 5%) which was significantly lower when compared to all other burial depths (TukeyHSD, *p* < 0.05).

### 2.4. Experiment 3—Prediction of Seed Longevity Using a CAT

**Figure 5:**
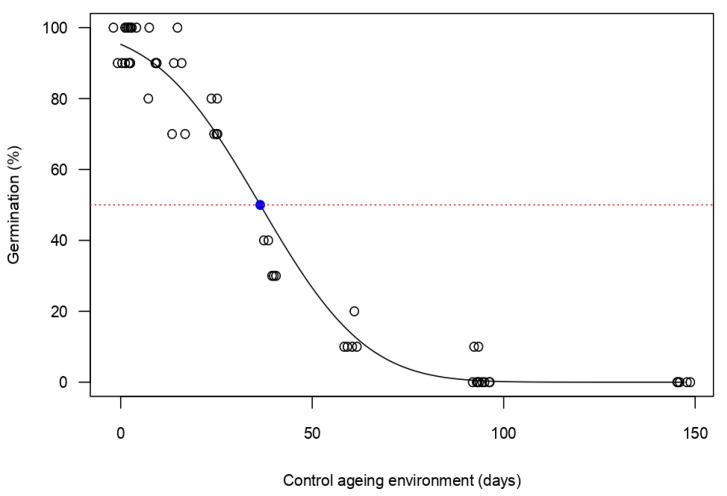
The response of a *S. platyphylla* seed lot under a controlled aging test run at 45 °C and 60% relative humidity. Upon sample retrieval from the test, the germinability was determined by incubation at 25/15 °C with a matching 12/12 h (light/dark) photoperiod. The blue dot indicates the predicted time when germination had fallen to 50% (P50). The red horizontal dashed line marks the 50% germination threshold. The open circles are the mean germination percentages of five replicate lots of 10 seeds, and overlapping germination values were slightly shifted to ensure all data points are visible.

The initial viability of the seed lot used was 98 ± 2%. As the aging test progressed, the germination percentage of the seed lot slowly reduced, before there was a rapid decline at around the 25th day (Figure 5). The fitted GLM model showed that the *S. platyphylla* seed lot lost about 50% of its viability (P50) within 36 days (Figure 5).

## 3. Discussion

### 3.1. Seed Germination Affected by Light and Temperature

Seed germination in *S. platyphylla* was strongly influenced by both temperature and light. The highest germination percentage (up to 99%) was recorded at a constant 21 °C under a 12/12 h light/dark photoperiod, while germination remained low in continuous darkness. This confirmed earlier findings by Flower [33], who also identified 21 °C as the optimal germination temperature for this species. Light has been widely recognized as a critical trigger for germination in aquatic plants [16]. For example, several *Carex* wetland species required up to eight hours of light exposure to achieve maximum germination [34], and light similarly enhanced germination in *Zostera marina* L. and various *Nymphaea* species [35,36,37]. The consistently high germination observed under both constant (21 °C) and all fluctuating temperature regimes tested (Figure 3 and Figure 4) suggested that *S. platyphylla* had a relatively broad thermal range for germination. However, germination appeared suppressed under colder (<15 °C) or hotter (>30 °C) conditions, implying that extreme seasonal temperatures may inhibit recruitment (Figure 2). Notably, alternating temperature regimes strongly promoted germination under light conditions even at relatively low mean temperatures (15/5 °C), whereas constant temperatures near these extremes inhibited germination. This pattern aligns with findings for *Phragmites australis* (Cav.) Trin. ex Steud. and *Typha latifolia* L., where final germination was sensitive to the amplitude of daily temperature fluctuations, and higher amplitudes significantly enhanced germination under light conditions [38]. Similarly, studies on *Carex* species from freshwater wetlands and mesic habitats showed that seeds incubated under alternating temperature regimes germinated more effectively than under constant temperatures [39], indicating that diurnal temperature fluctuations act as ecological cues for breaking dormancy and signaling favorable conditions for seedling establishment [40].

Comparable responses have been reported in other species within the Alismataceae family. For instance, *S. montevidensis* germinated optimally at 26 °C but failed to germinate at 10 °C [21], while *S. lancifolia* only reached full germination at 25 °C when exposed to light [20]. Under a 33/20 °C thermoperiod with a matching 14/10 h (light/dark) photoperiod, *S. platyphylla* achieved a reduced 62% germination [41]. This suggested that higher thermoperiods may exceed the optimal range for *S. platyphylla* germination. Given these temperature–light requirements, there are clear implications for management strategies. In cooler regions of Australia, such as Victoria, the southern and tableland regions of New South Wales (NSW), and parts of South Australia [42], natural winter conditions may delay germination. This offers an opportunity to schedule control actions (e.g., herbicide application or mechanical removal) shortly after germination but before seed set, ideally one to two months after the onset of warmer temperatures. In contrast, in warmer climates such as Queensland, northern NSW, and parts of Western Australia [42], germination may begin earlier and persist over a longer seasonal window. These regions may require more frequent monitoring and earlier control efforts, possibly starting in late winter or early spring.

Notably, some seeds demonstrated the ability to germinate in darkness, achieving ca. 37% germination at 21 °C (Figure 2). This indicated that while light typically enhances germination, it is not always essential, and temperature may serve as a more dominant cue in some cases. These differences might be attributed to physiological or genetic variation among seed lots, influenced by factors such as maternal environment, seed maturity, or seed age [43,44]. Similar environmentally responsive germination behaviors have been observed in other species. For instance, *Arabidopsis thaliana* (L.) Heynh. seeds produced under warmer conditions exhibit reduced dormancy [45], while seeds of *Thlaspi arvense* L. formed in shaded environments show reduced dependence on light for germination [46]. These patterns suggest a bet-hedging strategy, where variable germination responses increase the likelihood of successful establishment across fluctuating environmental conditions [47]. This highlighted the need for sustained, long-term control, as not all *S. platyphylla* seeds will respond uniformly to environmental triggers or treatment interventions. Very short light exposure during seed imbibition may have contributed to germination in the dark treatment; although overall germination was strongly light-dependent, stricter light exclusion could further refine assessments of true dark germination responses.

### 3.2. Seedling Emergence Affected by Burial Depth, and Seed Viability of Retrieved Seeds

Seedling emergence of *S. platyphylla* occurred only when seeds were placed on the substrate surface (Figure 4a), indicating that burial effectively inhibited germination. This is likely due to the absence of light reaching seeds beneath the surface, which aligns with findings from Experiment 1 where germination was significantly reduced in darkness. In aquatic habitats, light intensity is naturally reduced as it passes through the water column [48,49], further limiting the light available to buried seeds. Several aquatic and wetland species exhibit similar responses, showing reduced or no germination when seeds are buried. This has been observed in *Carex* spp. [34], *Myriophyllum spicatum* L., *Hydrocharias dubia* (Blume) Backer., *Ottelia alismoides* (L.) Pers., *Epilobium prostratum* Dougl. ex Hook. [50], *Vallisneria natans* (L.) H.B.K. [51], and *Zannichellia palustris* L. [52]. These findings suggest a widespread light requirement among aquatic macrophytes for successful germination.

The seeds used in this study were freshly harvested and likely exhibited strong dark-induced dormancy. It is well established that freshly shed seeds often have high levels of dormancy, which can be gradually alleviated through a process known as after-ripening [53]. This process leads to a progressive widening of the environmental conditions under which seeds can germinate, including the ability to germinate in darkness. For example, a small proportion of *Carex ferruginea* Scop. seeds buried for 3 to 15 months were able to germinate in darkness, although this species typically requires light [54]. Seeds buried under field conditions are known to continuously perceive environmental cues that regulate their dormancy status [55] and this dormancy cycling enables them to time their germination to coincide with favorable conditions [56,57]. Consequently, it is possible that *S. platyphylla* seeds, when buried over time, may gradually lose their dormancy and become capable of germinating even in the absence of light.

The high viability of seeds recovered after three months of burial (Figure 4b) suggests that burial may support seed persistence by protecting seeds until suitable germination conditions return. Seeds buried at 2.5 cm retained significantly higher viability than those at 0.5 cm, consistent with previous findings that deeper burial prolongs seed longevity. For example, a long-term study on arable weed seed survival revealed that seeds of *Avena fatua* L., *Polygonum aviculare* L., and *Orippa palustris* (L.) Besser buried at 15.0 cm maintained viability longer than those at shallower depths [58]. Although the present experiment only spanned three months, it demonstrated the potential for *S. platyphylla* to form a persistent soil seed bank. This highlights the importance of minimizing substrate disturbance, which could unearth viable seeds and expose them to light, potentially triggering germination. For example, in Victoria, spring and summer irrigation often involves refilling channels, which can disturb sediment and bring seeds back to the surface [59]. Likewise, mechanical or physical control efforts such as vegetation removal may also unintentionally expose buried seeds [60].

### 3.3. CAT to Estimate Seed Longevity of Sagittaria platyphylla

The results of the CAT indicated that *S. platyphylla* produces short-lived seeds (Figure 5), with an estimated field lifespan of one to less than three years (Table 1). Due to their short longevity, *S. platyphylla* seeds must germinate within a relatively narrow window of opportunity. To improve the likelihood of successful establishment, the species compensates by producing large quantities of seeds, increasing the chances that some will encounter favorable conditions for germination. A similar approach is seen in members of the Amaranthaceae family, which also produce numerous seeds with rapid germination responses to suit dynamic or unpredictable environments [61]. This life-history strategy is characteristic of ruderal (r-selected) species such as *S. platyphylla*, which tend to produce many small seeds, exhibit rapid growth, and are well-adapted for colonizing frequently disturbed habitats [62].

Seed persistence in the field is known to vary depending on a range of factors, including population origin, soil type [63], seed size [64,65], and disturbance in environments, and even between seed lots of the same species [24,66]. Additionally, seed aging is shaped by environmental conditions such as temperature, relative humidity, and oxygen availability, in combination with intrinsic seed traits like structural composition, chemical makeup, and desiccation tolerance [22,24]. Although no direct studies have quantified the seed longevity of *S. platyphylla* under natural conditions, some insight can be drawn from its close relative, *S. montevidensis*, which has been recorded to maintain seed viability for up to 3 years [33]. Among IAAPs, seed longevity spans a wide spectrum. For instance, seeds of *P. crassipes* can persist in the soil for up to 28 years [28], while *Glossostigma drummondii* Benth. and *Myriophyllum petreaum* Orchard produce short-lived seeds [30]. However, as discussed in Experiment 2, *S. platyphylla* seeds that remain buried for extended periods may gradually lose dormancy and eventually gain the ability to germinate in darkness. This reinforces the importance of managing the seedbank within a defined timeframe. From a practical standpoint, sustained management over a period of 3 to 4 years is likely to significantly reduce the viable SSB, thereby curbing population recovery. This is consistent with broader research indicating that preventing seed input over multiple consecutive seasons can reduce IAAP seedbanks to less than 5% of their original size [67]. Moreover, by reducing the SSB, this will create ecological niches for native species to re-establish, thereby restoring biodiversity and ecosystem function. Integrated approaches such as using herbicides on mature plants followed by treatments targeting seedlings can enhance effectiveness and speed up population decline.

## 4. Materials and Methods

### 4.1. Seed Collection

Seeds of *S. platyphylla* were randomly collected from an infestation in an urban creek in Sinnamon Park (27°32′42.6″ S; 152°56′40.9″ E), Southeast Queensland, Australia, during the peak flowering season (summer; October to February). The first seed lot, collected in late November, was used in Experiments 1 and 2, while the second lot, collected in early February, was used in Experiment 3.

Mature seeds, characterized by a subtle brown coloration, were easily released from the inflorescence by gentle touch. Seeds were collected in paper bags and immediately transferred to the seed laboratory at the University of Queensland (UQ) for cleaning of floral debris and removal of non-filled and immature seeds using a series of three stainless steel sieves (2.00, 1.00, and 0.5 mm; Endecotts Ltd., London, UK) applied sequentially. Non-filled and immature seeds were smaller and passed through the sieves, whereas most of the filled seeds were retained on the sieves. The cleaned seeds were kept in a dedicated seed storage room at 15 ± 1 °C and 15 ± 3% RH until being used for experimentation ca. 4 days later. Seeds not immediately needed were air-dried and then placed into a laboratory fridge at 4 ± 1 °C in a sealed container [68].

### 4.2. Seed Fill Determination

To determine the seed fill percentage of the seed lot prior germination test, seeds were examined using X-ray imaging (Faxitron MX-20, Lincolnshire, IL, USA). Four replicates of 25 seeds were randomly selected and exposed to 18 kV for 20 s, with images captured using Bioptics software. Based on the X-ray images, seeds were classified as filled and viable if they displayed a healthy embryo or as non-viable if they lacked an embryo or had an abnormally developed embryo. The percentage of filled seeds was calculated as the number of filled seeds relative to the total number of seeds in the sample.

### 4.3. Experiment 1—Effects of Light and Temperature on Seed Germination

Seeds were placed into 20 mL glass vials (Wheaton^®^ liquid scintillation vial, Millville, NJ, USA) filled with 15 mL distilled water and closed with its lid to minimize water evaporation and incubated at one of 10 constant temperatures ( 9, 11, 13, 15, 17, 19, 21, 23, 25, or 29 ± 1 °C) and under one of two illumination conditions (constant darkness or a 12/12 h light/dark photoperiod) on a thermogradient bar (Thermoline Ltd., Wetherill Park, Australia) to determine the optimal temperature for *S. platyphylla* germination. For the dark treatment, seeds were carefully handled to minimize or prevent light exposure. To maintain continuous darkness, half of the glass vials were quickly wrapped in two layers of aluminum foil after the seeds were imbibed. The remaining vials were exposed to cool white fluorescent light (with a light intensity of *ca*. 100 μmol m^−2^ s^−1^) to simulate a 12 h day period. A series of data loggers (Tinytags, TGP 4017, Hastings Data Loggers, Port Macquarie, NSW, Australia) was used to monitor temperature and RH in each of the 10 chambers on an hourly basis.

The experiment was conducted in a completely randomized design (CRD) with three replications of 25 seeds each. Prior to placing seeds into the glass vials, the seeds were shaken for 10 min in a 2% (*v*/*v*) sodium hypochlorite (NaOCl) solution (White King Bleach, Melbourne, Victoria, Australia) containing two drops of 1% Tween 20 (Labchem, Zelienople, PA 16063, USA), followed by a wash under running distilled water for 1 min to surface sterilize the seeds and prevent contamination during germination [69]. The effect of alternating temperature on germination was determined by undertaking a further germination study in three incubators (TRIL-750 Illuminated Refrigerator Incubator, Thermoline Ltd., Wetherill Park, Australia) set at either 15/5, 25/15, or 30/20 ± 2 °C with a 12/12 h (light/dark) photoperiod or under constant darkness. The experiment followed a randomized complete block design with four replications of 25 seeds. Seeds were surface-disinfected and placed in glass vials, as described in the previous thermogradient bar experiment.

Germination was assessed every second day and defined as the protrusion of the radicle by ≥2 mm through both the testa and fruiting layers. Once germinated, seedlings were removed from the glass vials and discarded. Seeds in the dark treatment were observed for germination inside a darkened tent and under a green safety light. The experiment was run for 28 days [16]. At the conclusion of both experiments, the ungerminated and healthy-looking seeds, as well as those free from contamination, were subjected to a second X-ray analysis to indirectly assess their viability. Filled but ungerminated seeds were defined as viable but dormant, whereas unfilled seeds were deemed to be non-viable.

### 4.4. Experiment 2—Effects of Burial Depth on Seedling Emergence and Viability of Retrieved Seeds

This experiment was undertaken in a CRD with four replications, each replication consisting of 10 seeds. Six-substrate burial depths were used (*viz.* 0—sown on the surface, 0.5, 1.0, 1.5, 2.0, and 2.5 cm deep). To do this, black plastic pots (300 mL) were filled with a substrate mixture (three parts of quartz sand and one part of peat) containing an Osmocote slow-release fertilizer (Osmocote^®^ Plus Trace Elements: Total All Purpose, Scots Co, Marysville, OH, USA). The fertilizer was added to the bottom of the pot at a rate of 3.75 g N kg^−1^ of the substrate mixture to supply macronutrients. The outside of the pot was marked to show the position to which the seeds would be buried. Ten seeds were placed inside a bag (16 cm^2^) made from 1.0 mm insect screen fiberglass mesh (Permastik™, Brisbane, QLD, Australia). The mesh bags helped in keeping the surface seeds in place and aided the retrieval of buried, ungerminated seeds at the termination of the experiment, while still permitting germinating seedlings to grow through the mesh. The mesh bags were buried at the designated depths, and the experiment ran for 90 days. To submerge the pots, a glass aquarium (216 L) was filled with a suitable culture solution following Smart and Barko [70] and kept constant at 25 ± 1 °C. The pH was adjusted to 6.5 ± 0.1 by using a carbon dioxide (CO_2_) injection system to create ideal growing conditions for any emerging seedlings. The incubation temperature of 25 °C had been determined to be optimal in Experiment 1. White light-emitting diode (LED) aquarium lights (Fluval, freshwater plant light, Denver, CO, USA), set at ca. 120 mmol m^−2^ s^−1^, were used to supply a 12/12 h (light/dark) photoperiod. The culture solution level was maintained at 40 cm above the level of the substrate surface in the pots. The seedling emergence was recorded weekly. After 90 days, all non-germinated seeds were retrieved and assessed for viability using a germination test in an incubator set at the optimum alternating temperature and lighting conditions, as determined in Experiment 1.

### 4.5. Experiment 3—Prediction of Seed Longevity Using a CAT

**Figure 6:**
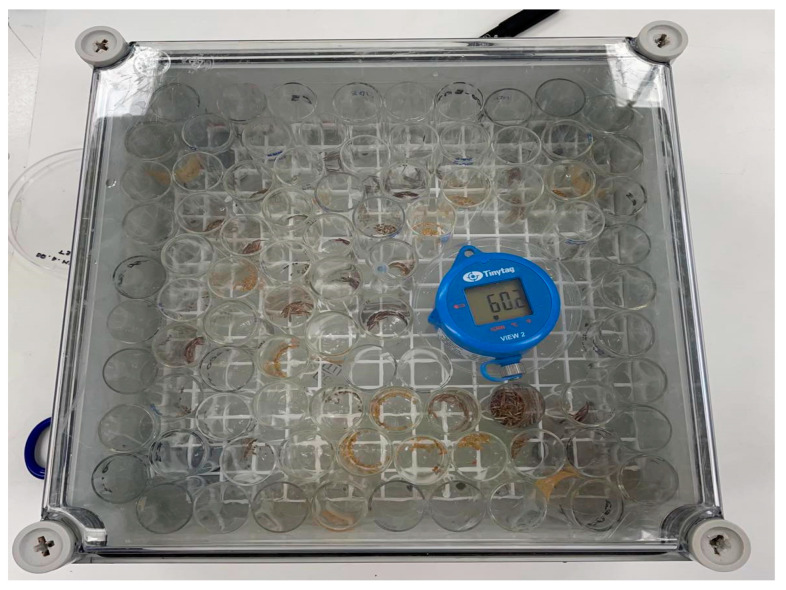
The airtight sealable chamber, containing lithium chloride to generate a 60.0% relative humidity environment, used to conduct the control aging test. Seeds in lots of 50 were placed into glass vials, then placed inside the box, and incubated in a laboratory oven set at 45 ± 1.0 °C, under constant dark conditions. Each glass vial was randomly removed at 0, 1, 3, 8, 15, 24, 39, 60, 92, 95, 120, and 147 days after treatment for the germination test of five replications with 10 seeds per replication.

Prior to the CAT experiment, the second seed lot was tested for germination under optimal conditions (25/15 °C, 12/12 h light/dark photoperiod) as identified in Experiment 1 with four replicates of 25 seeds per replicate. The CAT was conducted in the seed laboratory at UQ, Gatton Campus. Prior to transferring the seeds to the CAT-controlled environment, a rehydration step was applied to minimize damage caused by sudden rehydration [25]. To pre-equilibrate the seeds, 12 replicates of 50 freshly harvested seeds (ca. 10 days-old) were placed in open-top glass vials (25 mL) and exposed to a lithium chloride (LiCl) solution (370 g L^−1^ H_2_O), maintaining a RH of 47 ± 1% inside a sealable chamber, incubated at 20 ± 1 °C. The sealable chamber had a plastic grid stand inside to keep the glass vials with seeds above the LiCl solution (Figure 6). After 14 days of seed rehydration, all 12 glass vials containing seeds were transferred into a second identical sealable chamber containing a LiCl solution (300 g L^−1^ H_2_O), producing a 60 ± 2% RH. The box was then placed into a laboratory oven set at 45.0 ± 0.5 °C to undergo the CAT under darkness. As the RH produced by the LiCl solution inside the box is known to decrease with time, distilled water was added weekly to readjust the RH back to 60% [25,71]. Glass vials were removed periodically (at 0, 1, 3, 8, 15, 24, 39, 60, 92, 95, 120, and 147 days after treatment), and their seeds were assessed for viability using a germination test. The germination test was conducted under optimal temperature and light conditions, as identified in Experiment 1, with five replications of 10 seeds per replicate, taken at random from each glass vial.

### 4.6. Data Collection and Analysis

All data analyses were carried out in R version 4.2.2 using R-studio version 2023.09.1 + 494 [72,73]. Germination percentage was calculated using the method of Davies, Di Sacco, and Newton [69] and following adjustment for seed fill using a modification of the method of Merritt and Rokich [74].

Equation (1): Germination percentage
  (1) $$Germination(\%)=\frac{Total\;seeds\;germinated}{Total\;seeds\;per\;replicate}\times 100$$
Equation (2): Final germination percentage adjusted by seed fill
  (2) $$Final\;germination(\%)=\frac{Germination(\%)}{Seed\;fill(\%)}\times 100$$

To all germination data in Experiment 1, a GLM was fitted with a logistic link function and a binominal error to analyze the germination count data [75,76]. For the burial depth in Experiment 2, a GLM with the same binominal approach was also used to analyze seedling emergence to test the effect of different burial depths. The significance of model terms was assessed using Type II ANOVA with the “car” package [77]. Then a Tukey post hoc test using the “multcomp” package [78] was applied to compare the differences between the means of treatment in both experiments.

To estimate seed longevity in Experiment 3, a GLM model with a probit link function and binomial error was fitted to the seed longevity data. The response variable was final germination, with days in the CAT as explanatory variable [79]. The time taken for germination to decline by 50% in the CAT environment (P_50_) was estimated by the fitted GLM model. Therefore, viability was calculated using Equation 3, developed by Ellis and Roberts [80].

Equation (3): Seed longevity
  (3) v = Ki − (p/σ)
  where v is the viability of seeds after p days in the aging environment, Ki represents the initial probit viability of the seed lot, and σ is the time taken for viability to decrease by one normal equivalent deviate, which is equivalent to the standard deviation of the frequency distribution of seed deaths over time (days). Seed classification based on the predicted P_50_ value from the model was determined as shown below (Table 2).

## 5. Conclusions

This study demonstrated that *S. platyphylla* seeds germinate optimally at 21 °C, with successful emergence across a wide temperature range (17 to 29 °C) under a 12/12 h (light/dark) photoperiod. High germination also occurred under fluctuating temperature regimes (15/5 °C, 25/15 °C, and 30/20 °C), indicating adaptability to variable natural conditions. A strong light requirement means that seeds buried even at shallow depths of 0.5 cm failed to germinate, although they remained viable for at least 1 to 3 years, highlighting short-term persistence in the seedbank. Practically, these findings suggest that minimizing sediment disturbance to keep seeds buried, combined with timing control interventions around seasonal temperature windows, can effectively reduce *S. platyphylla* recruitment and contribute to more strategic weed management.

Given these findings, future research should focus on how local habitat characteristics, such as sediment type, hydrology, and canopy cover, influence seed persistence and germination potential. Studies examining the interactive effects of sediment disturbance, mechanical control, and herbicide application on seedbank dynamics are urgently needed. Such work will be essential for developing more effective and integrated management strategies that not only target above-ground plant biomass but also address the regenerative potential held within the seedbank.

## Figures and Tables

**Table 1:** Type II ANOVA table (analysis of deviance) from a Generalized Linear Model of seed germination under different light conditions and temperatures.

Term	Likelihood Ratio Chi-Square	Df	*p*-Value
Light conditions (light vs. dark)	289.95	1	<2.2 × 10^−16^
Temperature (10 levels)	641.58	9	<2.2 × 10^−16^
Light condition x temperature	82.39	9	5.40 × 10^−14^
Residuals	-	40	-
AIC (Akaike Information Criterion)	-	-	311.86

**Table 2:** The time for germination declining to 50% (P_50_) in the controlled aging test (CAT) was used to classified the seeds into longevity categories and given a predicted lifespan under field conditions [25].

P50 Value (Days)	P50 Category	Lifespan Predicted In Situ (Years)
<20	Transient	<1
20 < P50 < 50	Short-lived	1 to 3
>50	Long-lived	>3

## Data Availability

The original contributions presented in this study are included in the article. Further inquiries can be directed at the corresponding author.

## Abbreviations

The following abbreviations are used in this manuscript:

IAAP	Invasive alien aquatic plant
SSB	Substrate seedbank
CAT	Controlled aging test
RH	Relative humidity
GLM	Generalized linear model
CRD	Completely randomized design
AIC	Akaike information criterion
P50	The time taken for germination to decline to 50%
NSW	New South Wales
UQ	University of Queensland
NaOCl	Sodium hypochlorite
CO2	Carbon dioxide
LED	Light-emitting diode
LiCl	Lithium chloride

## References

[1] Adair R.J., Keener B.R., Kwong R.M., Sagliocco J.L., Flower G.E. (2012). The biology of Australian weeds 60. Sagittaria platyphylla (Engelmann) J.G. Smith and Sagittaria calycina Engelmann. Plant Prot. Q..

[2] Clements D., Dugdale T., Kwong R. (2018). Developing Best Practice Management Strategies for Sagittaria in Australia. Phase 1: Current Management Practices—May 2018.

[3] Kwong R.M. Is delta arrowhead (Sagittaria platyphylla) a suitable target for biological control in Australia? In Proceedings of the 19th Australasian Weeds Conference, “Science, Community and Food Security: The Weed Challenge”, Hobart, Australia, 1–4 September 2014.

[4] Dugdale T.M., Kwong R.M. (2023). National Best Practice Management Manual for Sagittaria (Sagittaria platyphylla).

[5] Kwong R.M., Sagliocco J.L., Harms N.E., Butler K.L., Martin G.D., Green P.T. (2019). Could enemy release explain invasion success of Sagittaria platyphylla in Australia and South Africa?. Aquat. Bot..

[6] Kwong R.M., Sagliocco J.L., Harms N.E., Butler K.L., Green P.T., Martin G.D. (2017). Biogeographical comparison of the emergent macrophyte, *Sagittaria platyphylla* in its native and introduced ranges. Aquat. Bot..

[7] Robledo M., Contreras S., Johanna B., Galli C. (2021). First Miocene megafossil of arrowhead, alismataceous plant Sagittaria, from South America. Acta Palaeontol. Pol..

[8] Cohen O., Riov J., Katan J., Gamliel A., Bar P. (2008). Reducing persistent seed banks of invasive plants by soil solarization: The case of *Acacia saligna*. Weed Sci..

[9] Zimdahl R.L., Zimdahl R.L. (2018). Chapter 22—Weed-management systems. Fundamentals of Weed Science.

[10] Bicalho E.M. (2024). Soil seed banks, persistence and recruitment: Memories of a partially non-lived life?. Theor. Exp. Plant Physiol..

[11] Gioria M., Carta A., Baskin C.C., Dawson W., Essl F., Kreft H., Pergl J., van Kleunen M., Weigelt P., Winter M. (2021). Persistent soil seed banks promote naturalisation and invasiveness in flowering plants. Ecol. Lett..

[12] Biswas P.S., Rashid M.M., Khatun H., Yasmeen R., Biswas J.K., Hasanuzzaman M., Fujita M., Nahar K., Biswas J.K. (2019). Chapter 11—Scope and progress of rice research harnessing cold tolerance. Advances in Rice Research for Abiotic Stress Tolerance.

[13] Delaisse C., Yeoh P.B., Didham R.K., Lewandrowski W., Scott J.K., Webber B.L. (2023). Improving weed management by targeting the seed ecology of blackberry (*Rubus anglocandicans*) in a biodiversity hotspot. Aust. J. Bot..

[14] Chauhan B.S., Johnson D.E. (2010). The role of seed ecology in improving weed management strategies in the tropics. Advances in Agronomy.

[15] Dey P., Pratap T., Mishra S., Pandit P. (2018). Weed seed bank in soil as affected by different weed management practices in spring sweet corn. Indian J. Weed Sci..

[16] Baskin C.C., Baskin J.M. (2014). Germination ecology of plants with specialized life cycles and/or habitats. Seeds: Ecology, Biogeography, and Evolution of Dormancy and Germination.

[17] Crocker W. (1907). Germination of seeds of water plants. Bot. Gaz..

[18] USDA Plant Factsheet: Duck Potato (Sagittaria latifolia Willd). https://plants.usda.gov/home/plantProfile?symbol=SALA.

[19] Ozaki Y., Shimono Y., Tominaga T. (2018). Germination characteristics of *Sagittaria trifolia*. Weed Biol. Manag..

[20] Gordon C.E., Velasquez J. (1989). Dispersion, germination and growth of seedlings of *Sagittaria lancifolia* L.. Folia Geobot. Phytotax..

[21] Pitol A., Cechin J., Schreiber F., Santos Moisinho I., Andres A., Agostinetto D. (2022). Ecophysiological aspects of seed germination in *Sagittaria montevidensis* biotypes resistant and susceptible to herbicides. Pesqui. Agropec. Bras..

[22] Ballesteros D., Pritchard H.W., Walters C. (2020). Dry architecture: Towards the understanding of the variation of longevity in desiccation-tolerant germplasm. Seed Sci. Res..

[23] Brock M.A. (2011). Persistence of seed banks in Australian temporary wetlands. Freshw. Biol..

[24] Nadarajan J., Walters C., Pritchard H.W., Ballesteros D., Colville L. (2023). Seed longevity-the evolution of knowledge and a conceptual framework. Plants.

[25] Long R.L., Panetta F.D., Steadman K.J., Probert R., Bekker R.M., Brooks S., Adkins S.W. (2008). Seed persistence in the field may be predicted by laboratory-controlled aging. Weed Sci..

[26] Roberts E.H., Roberts E.H. (1972). Storage Environment and the Control of Viability. Viability of Seeds.

[27] Li W. (2014). Environmental opportunities and constraints in the reproduction and dispersal of aquatic plants. Aquat. Bot..

[28] Sullivan P.R., Wood R. Water hyacinth (*Eichhornia crassipes* (Mart.) Solms) seed longevity and the implications for management. Proceedings of the Eighteenth Australasian Weeds Conference.

[29] Panetta F.D. (2010). Seed persistence of the invasive aquatic plant, *Gymnocoronis spilanthoides* (Asteraceae). Aust. J. Bot..

[30] Tuckett R.E., Merritt D.J., Hay F.R., Hopper S.D., Dixon K.W. (2010). Comparative longevity and low-temperature storage of seeds of Hydatellaceae and temporary pool species of south-west Australia. Aust. J. Bot..

[31] Karrer G., Lehner F., Waldhaeuser N., Knolmajer B., Hall R.M., Poór J., Jócsák I., Kazinczi G. (2024). Long-term seed survival of common ragweed (*Ambrosia artemisiifolia* L.) after burial. NeoBiota.

[32] Moravcová L., Carta A., Pyšek P., Skálová H., Gioria M. (2022). Long-term seed burial reveals differences in the seed-banking strategies of naturalized and invasive alien herbs. Sci. Rep..

[33] Flower G.E. (2003). The Biology and Ecology of Arrowhead (Sagittaria montevidensis Cham. et Schlecht), A Weed in Rice in NSW.

[34] Kettenring K.M., Gardner G., Galatowitsch S.M. (2006). Effect of light on seed germination of eight wetland *Carex* species. Ann. Bot..

[35] Dalziell E.L., Funnekotter B., Mancera R.L., Merritt D.J. (2019). Seed storage behaviour of tropical members of the aquatic basal angiosperm genus *Nymphaea* L. (Nymphaeaceae). Conserv. Physiol..

[36] Kadono Y. (1982). Germination of the turion of *Potamogeton crispus* L.. Physiol. Ecol. Jpn..

[37] Moore K.A., Orth R.J., Nowak J.F. (1993). Environmental regulation of seed germination in *Zostera marina* L. (eelgrass) in Chesapeake Bay: Effects of light, oxygen and sediment burial. Aquat. Bot..

[38] Ekstam B., Forseby Å. (1999). Germination response of *Phragmites australis* and *Typha latifolia* to diurnal fluctuations in temperature. Seed Sci. Res..

[39] Schütz W. (1999). Germination responses of temperate Carex-species to diurnally fluctuating temperatures—A comparative study. Flora.

[40] Liu K., Baskin J.M., Baskin C.C., Bu H., Du G., Ma M. (2013). Effect of Diurnal Fluctuating versus Constant Temperatures on Germination of 445 Species from the Eastern Tibet Plateau. PLoS ONE.

[41] Zou T.T., Lyu S.T., Jiang Q.L., Shang S.H., Wang X.F. (2023). Pre- and post-pollination barriers between two exotic and five native *Sagittaria* species: Implications for species conservation. Plant Divers..

[42] Australian Government Climate Classification Maps. http://www.bom.gov.au/climate/maps/averages/climate-classification/.

[43] Née G., Xiang Y., Soppe W.J.J. (2017). The release of dormancy, a wake-up call for seeds to germinate. Curr. Opin. Plant Biol..

[44] Penfield S., MacGregor D.R. (2016). Effects of environmental variation during seed production on seed dormancy and germination. J. Exp. Bot..

[45] Kerdaffrec E., Nordborg M. (2017). The maternal environment interacts with genetic variation in regulating seed dormancy in Swedish *Arabidopsis thaliana*. PLoS ONE.

[46] Chen D., Yuan Z., Wei Z., Hu X. (2022). Effect of maternal environment on seed germination and seed yield components of *Thlaspi arvense*. Ind. Crops Prod..

[47] Sharma E., Majee M. (2023). Seed germination variability: Why do genetically identical seeds not germinate at the same time?. J. Exp. Bot..

[48] Bliss D., Smith H. (1985). Penetration of light into soil and its role in the control of seed germination. Plant Cell Environ..

[49] Lythgoe J.N. (1988). Light and vision in the aquatic environment. Proceedings of the Sensory Biology of Aquatic Animals.

[50] Xiao C., Xing W., Liu G. (2010). Seed germination of 14 wetland species in response to duration of cold-wet stratification and outdoor burial depth. Aquat. Biol..

[51] Ke X., Li W. (2006). Germination requirement of *Vallisneria natans* seeds: Implications for restoration in Chinese lakes. Hydrobiologia.

[52] Spencer D.F., Ksander G.G. (2002). Sedimentation disrupts natural regeneration of *Zannichellia palustris* in Fall River, California. Aquat. Bot..

[53] Chahtane H., Kim W., Lopez-Molina L. (2016). Primary seed dormancy: A temporally multilayered riddle waiting to be unlocked. J. Exp. Bot..

[54] Schütz W. (2002). Dormancy characteristics and germination timing in two alpine *Carex* species. Basic Appl. Ecol..

[55] Finch-Savage W.E., Leubner-Metzger G. (2006). Seed dormancy and the control of germination. New Phytol..

[56] Finch-Savage W.E., Footitt S. (2017). Seed dormancy cycling and the regulation of dormancy mechanisms to time germination in variable field environments. J. Exp. Bot..

[57] Lamont B.B., Pausas J.G. (2023). Seed dormancy revisited: Dormancy-release pathways and environmental interactions. Funct. Ecol..

[58] Conn J.S., Beattie K.L., Blanchard A. (2006). Seed viability and dormancy of 17 weed species after 19.7 years of burial in Alaska. Weed Sci..

[59] Victoria Government Regional Irrigated Land and Water Use Mapping Program. https://www.water.vic.gov.au/our-programs/regional-irrigated-land-and-water-use-mapping-program.

[60] Thiemer K., Schneider S.C., Demars B.O.L. (2021). Mechanical removal of macrophytes in freshwater ecosystems: Implications for ecosystem structure and function. Sci. Total Environ..

[61] Kadereit G., Newton R.J., Vandelook F. (2017). Evolutionary ecology of fast seed germination: A case study in Amaranthaceae/Chenopodiaceae. Perspect. Plant Ecol. Evol. Syst..

[62] Rejmanek M., Richardson D.M. (1996). What Attributes Make Some Plant Species More Invasive?. Ecology.

[63] Moles A.T., Warton D.I., Westoby M. (2003). Seed size and survival in the soil in arid Australia. Austral Ecol..

[64] Bekker R.M., Bakker J.P., Grandin U., Kalamees R., Milberg P., Poschlod P., Thompson K., Willems J.H. (1998). Seed size, shape and vertical distribution in the soil: Indicators of seed longevity. Funct. Ecol..

[65] Ghersa C.M., Martínez-Ghersa M.A. (2000). Ecological correlates of weed seed size and persistence in the soil under different tilling systems: Implications for weed management. Field Crops Res..

[66] Rieks D.K., Lloyd F. (2005). Wet heat as a mechanism for dormancy release and germination of seeds with physical dormancy. Weed Sci..

[67] Menalled F., Schonbeck M. Manage the Weed Seed Bank—Minimize “Deposits” and Maximize “Withdrawals”. https://eorganic.org/node/2806.

[68] Delesalle V.A., Blum S. (1994). Variation in germination and survival among families of *Sagittaria latifolia* in response to salinity and temperature. Int. J. Plant Sci..

[69] Davies R., Di Sacco A., Newton R. (2015). Germination Testing: Procedures and Evaluation.

[70] Smart M.R., Barko J.W. (1985). Laboratory culture of submersed freshwater macrophytes on natural sediments. Aquat. Bot..

[71] Royal Botanic Gardens Kew (2022). MSBP Technical Information Sheet 01—Comparative Longevity.

[72] Posit Team (2023). RStudio: Integrated Development Environment for R (Version 2023.09.1+494) [Computer Software].

[73] RStudio Team (2023). RStudio: Integrated Development for R (Version 4.2.2) [Computer Software].

[74] Merritt D., Rokich D., Sweedman L., David M. (2006). Seed biology and ecology. Australian Seeds: A Guide to Their Collection, Identification and Biology.

[75] Carvalho F.J., Santana D.G., Araújo L.B. (2018). Why analyze germination experiments using Generalized Linear Models?. J. Seed Sci..

[76] Gianinetti A. (2020). Basic features of the analysis of germination data with generalized linear mixed models. Data.

[77] Fox J., Weisberg S. An R Companion to Applied Regression. https://www.john-fox.ca/Companion/.

[78] Hothorn T., Bretz F., Westfall P. (2008). Simultaneous inference in general parametric models. Biometr. J..

[79] Mondoni A., Orsenigo S., Donà M., Balestrazzi A., Probert R., Hay F., Petraglia A., Abeli T. (2014). Environmentally induced transgenerational changes in seed longevity: Maternal and genetic influence. Ann. Bot..

[80] Ellis R.H., Roberts E.H. (1980). Improved equations for the prediction of seed longevity. Ann. Bot..

